# Selective Activation of Cancer Stem Cells by Size-Specific Hyaluronan in Head and Neck Cancer

**DOI:** 10.1155/2015/989070

**Published:** 2015-09-10

**Authors:** Marisa Shiina, Lilly Y. W. Bourguignon

**Affiliations:** San Francisco Veterans Affairs Medical Center and Department of Medicine, University of California at San Francisco and Endocrine Unit (111N2), 4150 Clement Street, San Francisco, CA 94121, USA

## Abstract

We determined that human head and neck cancer cells (HSC-3 cell line) contain a subpopulation displaying cancer stem cell (CSC) properties and are very tumorigenic. Specifically, we investigated whether different sizes of hyaluronan (HA) (e.g., 5 kDa, 20 kDa, 200 kDa, or 700 kDa-HA-sizes) play a role in regulating these CSCs. First, we observed that 200 kDa-HA (but not other sizes of HA) preferentially induces certain stem cell marker expression resulting in self-renewal and clonal formation of these cells. Further analyses indicate that 200 kDa-HA selectively stimulates the expression of a panel of microRNAs (most noticeably miR-10b) in these CSCs. Survival protein (cIAP-1) expression was also stimulated by 200 kDa-HA in these CSCs leading to cisplatin resistance. Furthermore, our results indicate that the anti-miR-10 inhibitor not only decreases survival protein expression, but also increases chemosensitivity of the 200 kDa-HA-treated CSCs. These findings strongly support the contention that 200 kDa-HA plays a pivotal role in miR-10 production leading to survival protein upregulation and chemoresistance in CSCs. Together, our findings suggest that selective activation of oncogenic signaling by certain sizes of HA (e.g., 200 kDa-HA) may be instrumental in the formation of CSC functions leading to tumor cell survival and chemoresistance in head and neck cancer progression.

## 1. Introduction

Human head and neck squamous cell carcinoma (HNSCC) is the sixth most common cancer worldwide and is also one of the most deadly cancers [1]. The three-year survival rate for patients with advanced-stage HNSCC and treated with standard therapy is only 30 to 50% [1]. This deadly disease includes cancers of the lip, oral cavity, pharynx, hypopharynx, larynx, nose, nasal, sinuses, neck, ears, and salivary glands [1]. Nearly 40 to 60% of HNSCC patients subsequently develop recurrences or distant metastases [1]. Thus, there is currently a great need to clarify the key mechanisms of tumor initiation and progression underlying the clinical behavior of HNSCC.

Accumulating evidence indicates that most tumors contain a small population of cells which persistently initiate tumor growth and promote tumor progression. These “cancer stem cells (CSCs)” [also called “tumor-initiating cells (TICs)”] share several of the hallmarks of normal stem cells [2, 3]. For example, CSCs undergo self-renewal, maintain quiescence, display multipotentiality, and express survival protein/antiapoptosis proteins [2, 3]. Another well-known property of CSCs is their ability to expand the stem cell population by undergoing cell proliferation/survival and/or clone formation and differentiation [2, 3]. A number of studies have identified specific molecules expressed in CSCs that correlate with both stem cell properties and tumor cell behaviors. Among such molecules is CD44 which is a multifunctional transmembrane glycoprotein expressed in many cells and tissues including HNSCC cells and other carcinoma tissues [2, 3]. CD44 is commonly expressed in various isoforms generated by alternative mRNA splicing of variant exons inserted into an extracellular membrane-proximal site [4]. CD44 is expressed in both normal and cancer stem cells (CSCs) and serves as an important stem cell marker [2, 3].

Hyaluronan (HA) is a major component in the extracellular matrix (ECM) of most mammalian tissues. HA is a nonsulfated, unbranched glycosaminoglycan consisting of repeating disaccharide units, D-glucuronic acid, and N-acetyl-D-glucosamine [5, 6]. Under physiological conditions, HA is synthesized by several HA synthases [7] and HA fragments of low molecular mass are produced by hyaluronidases or oxidation [8]. One general concept which has emerged from these studies is that HA fragments (small- versus mid-size-HAs) and their larger precursor molecules (i.e., intact HA) may be involved in distinct biological activities [9, 10]. In addition, the formation of biologically active HA fragments from the large HA in the ECM occurs during periods of proliferation, migration, differentiation, and development as well as injury-related repairs [9, 10]. A number of studies indicate that large size-HA promotes transcriptional activation and differentiation, whereas small-size-HA induces cell proliferation and migration [9, 10]. HA is enriched in many types of tumors [11, 12] and also has been found to be increased in stem cell niches [13, 14]. Furthermore, the unique HA-enriched microenvironment appears to be involved in both self-renewal and differentiation of normal human stem cells [13, 14].

All CD44 isoforms contain a HA-binding site in their extracellular domain and thereby serve as a major cell surface receptor for HA [5, 6]. The fact that both CD44 and HA are overexpressed at tumor attachment sites and that HA binding to CD44 stimulates a variety of tumor cell-specific functions and tumor progression [11, 12] suggests that the HA-CD44 interaction is a critical requirement for tumor progression. However, the cellular and molecular mechanisms underlying HA's ability to regulate CD44-positive CSCs by different sizes of HA during HNSCC progression remain poorly understood. Furthermore, the oncogenic mechanism(s) occurring during activation of CSCs by size-specific HAs during head and neck cancer progression remain(s) to be unknown.

In this study we have investigated the effects of different sizes of HA (ranging from 5 kDa to 700 kDa) on CSC signaling and function in head and neck cancer cells. Our results indicate that 200 kDa-HA (and to a much lesser extent 5 kDa-HA, 20 kDa-HA, and 700 kDa-HA) plays an important role in the selective activation of pluripotency factor (stem cell marker) expression, microRNA signaling and CSC functions required for tumor cell behaviors lead to head and neck cancer progression.

## 2. Materials and Methods

### 2.1. Cell Culture

Tumor-derived HSC-3 cell line (from human squamous carcinoma cells of mouth), was kindly provided from Dr. Randy Kramer (University of California, San Francisco, CA). Cells were grown in DMEM/F12 medium (Corning, NY) supplemented with 10% fetal bovine serum.

### 2.2. Antibodies and Reagents

Rabbit anti-CD44v3 antibody was obtained from EMD Chemicals (Gibbstown, NJ). Other immunoreagents such as rabbit anti-cIAP1 antibody were from Abcam (Cambridge, MA) and rabbit anti-actin antibody was purchased from Cell Signaling (Beverly, MA), respectively. Cisplatin was obtained from Millipore (Darmstadt, Germany). Different sizes of research grade HA fragments (e.g., 5 kD, 20 kDa, 200 kDa, and 700 kDa) were purchased from Lifecore Biomedical (Chaska, MN).

### 2.3. Sorting Tumor-Derived HSC-3 Cell Populations by Multicolor Fluorescence-Activated Cell Sorter (FACS)

The identification of aldehyde dehydrogenase-1 (ALDH1) activity from tumor-derived HSC-3 cells was conducted using the ALDEFLUOR kit (StemCell Technologies, Durham, NC). Specifically, tumor cells were suspended in ALDEFLUOR assay buffer containing ALDH1 substrate (BAAA, 1 mol/L per 1 × 10^6^ cells) and incubated for 30 min at 37°C. As a negative control, HSC-3 cells were treated with a specific ALDH1 inhibitor, 50 mmol/L diethylaminobenzaldehyde (DEAB) (50 mmol/L).

Next, for labeling cell surface marker, tumor-derived HSC-3 cells were resuspended in 100 *μ*L ALDEFLUOR buffer followed by incubating with 20 *μ*L allophycocyanin- (APC-) labeled anti-CD44v3 antibody (recognizing the v3-specific domain of CD44) or APC-labeled normal mouse IgG (as a control) (BD Bioscience, San Jose, CA) for 15 min at 4°C. For FACS sorting, tumor cells were resuspended in PBS buffer followed by FACS (BD FACS Aria llu, BD Bioscience, San Jose, CA) sorting using dual-wavelength analysis. Subsequently, CD44v3^high^ALDH1^high^ tumor cell population was collected and used for various experiments described in this study.

### 2.4. Tumorgenicity Assay

Nonobese, diabetic/severe combined immunodeficient (NOD/SCID) immunocompromised mice (5-week-old female mice) were purchased from Charles River Laboratories International, Inc. (Wilmington, MA) and maintained in microisolator cages. Specifically, these NOD/SCID were injected subcutaneously and/or submucosally in the floor of the mouth with sorted CD44v3^high^ALDH1^high^ cells or unsorted HSC-3 cells (suspended in 0.1 mL Matrigel Basement Membrane Matrix) ranging from 50, 500, to 5,000 cells. These mice were then monitored twice weekly for palpable tumor formation and euthanized 4 or 8 weeks after transplantation to assess tumor formation. Tumors were measured using a Vernier caliper, weight, and photographed. A portion of the subcutaneous tumors and/or submucosa tumors was collected. Some tumors were cut into small fragments with sterile scissors and miced with a sterile scalpel, rinsed with Hans' balanced salt solution containing 2% heat-inactivated calf serum (Invitrogen), and centrifuged for 5 min at 1,000 rpm. The resulting tissue specimen was placed in a solution of DMED F-12 containing 300 U/mL collagenase and 100 U/mL hyaluronidase (StemCell Technologies, Durham, NC). The mixture was incubated at 37°C to dissociate cells. The digestion was arrested with the addition of FBS and the cells were filtered through a 40 *μ*m nylon sieve. The cells were washed twice with Hans' balanced salt solution plus 2% heat-inactivated calf serum for FACS as described above.

### 2.5. Quantitative PCR (Q-PCR)

Total RNA was isolated from CD44v3^high^ALDH1^high^ cells (pretreated with no HA or 5 kDa-HA or 20 kDa-HA or 200 kDa-HA or 700 kDa-HA for 24 h) using Tripure Isolation Reagent kits (Roche Applied Science, Indianapolis, IN). First-stranded cDNAs were synthesized from RNA using Superscript First-Strand Synthesis system (Invitrogen, Carlsbad, CA). Gene expression was quantified using probe-based Sybr Green PCR Master Mix kits, ABI PRISM 7900HT sequence detection system, and SDS software (Applied Biosystems, Foster City, CA). A cycle threshold (minimal PCR cycles required for generating a fluorescent signal exceeding a preset threshold) was determined for each gene of interest and normalized to a cycle threshold for a housekeeping gene (36B4) determined in parallel. The 36B4 is a human acidic ribosomal phosphoprotein PO whose expression was not changed in tumor cells. The Q-PCR primers used for detecting gene expression of Oct4, Sox2, Nanog, and KLF-4 were as follows: specifically, two Oct4-specific primers (the sense primer 5′-GGTATTCAGCCAAACGACCA-3′ and the antisense primer 5′-CACACTCGGACCACATCCTT-3′); two Sox2-specific primers (the sense primer 5′-GACAGTTACGCGCACATGAA-3′ and the antisense primer 5′-TAGGTCTGCGAGCTGGTCAT-3′); and two Nanog-specific primers (the sense primer 5′-GTGATTTGTGGGCCTGAAGA-3′ and the antisense primer 5′-ACACAGCTGGGTGGAAGAGA-3′); two KLF4-specific primers (the sense primer 5′-CACCATGGACCCGGGCGTGGCTGCCAGAAA and the antisense primer 5′-TTAGGCTGTTCTTTTCCGGGGCCACGA) were used. The Q-PCR primers used for detecting gene expression of various miRNAS were as follows: specifically, two miR-10b-specific primers (the sense primer 5′-GGATACCCTGTAGAACCGAA and the antisense primer 5′-CAGTGCGTGTCGTGGAGT); two 23b-27b-specific primers (the sense primer 5′-TCACATTGCCAGGGATTACCA and the antisense primer 5′-TGCACCTGTTCTCCAATCTGC); two miR-373 primers (the sense primer 5′-CCTTCAACAGCTCATCAAGGGCT and the antisense primer 5′-TACCCGCCCCCTCACCCAATCAA); two miR-34a primers (the sense primer 5′-TGGCAGTGTCTTAGCTGGTTG and the antisense primer 5′-GGCAGTATACTTGCTGATTGCTT); two miR-145 primers (the sense primer 5′-GGTCCAGTTTTCCCAGG and the antisense primer 5′-CAGTGCGTGTCGTGGAGT); two miR-181a (the sense primer 5′-AACATTCAACGCTGTCGGT and the antisense primer 5′CAGTCAACGGTCAGTGGTTT). Finally, for detecting 36B4 gene expression, two 36B4-specific primers (the sense primer 5′-GCGACCTGGAAGTCCAACTAC-3′ and the antisense primer 5′-ATCTGCTGCATCTGCTTGG-3′) were used.

### 2.6. Immunoblotting Techniques

RIPA buffer-solubilized cell lysate of untransfected CD44v3^high^ALDH1^high^ cells (pretreated with no HA or 5 kDa-HA or 20 kDa-HA or 200 kDa-HA for 3 days) or CD44v3^high^ALDH1^high^ cells transfected with anti-miR-10b inhibitor or miRNA-negative control followed by 200 kDa-HA (50 *μ*g/mL) addition (or no HA addition) for 3 days at 37°C were immunoblotted using various immunoreagents [e.g., mouse anti-cIAP-1 (2 *μ*g/mL) or goat anti-actin (2 *μ*g/mL) (as a loading control), resp.].

### 2.7. Spheroid Formation and Self-Renewal Assays

CD44v3^high^ALDH1^high^ cells were analyzed for spheroid formation and self-renewal using the methods described previously [15].

### 2.8. Clone Formation and Differentiation Assays

For analyzing the clone formation (differentiation) properties, CD44v3^high^ALDH1^high^ cells (treated with 200 kDa-HA or no HA) were dissociated from spheres and inoculated to 6-well plates at a density of 200 cells per well in six-well plates, and cultured with DMEM/F12 plus 10% serum for ~21 days. After most cell clones expand to >50–100 cells, they were fixed with methanol followed by staining with crystal violet. The clone formation efficiency (CFE) was expressed as the ratio of the clone number to the planted cell number.

### 2.9. Tumor Cell Growth Assays

Sphere-derived CD44v3^high^ALDH1^high^ cells (5 × 10^3^ cells/well) were incubated in basal medium with B27 plus 200 kDa-HA (50 *μ*g/mL) or no HA. Medium were replenished every 3 days. Twenty-on (21) days after plating, total cell growth was then counted under a microscope at low magnification.

In some cases, these sphere-derived CD44v3^high^ALDH1^high^ cells (transfected with anti-10b inhibitor or miRNA-negative control) were also incubated with various concentrations of cisplatin (0–20 *μ*M) with no HA or with 200 kDa-HA (50 ug/mL). After 7-day incubation at 37°C, CellTiter-Glo Luminescent Cell Viability Assays (Promega, Madison, WI) were analyzed as described previously [15]. The percentage of absorbance relative to untreated controls (i.e., cells treated with neither HA nor chemotherapeutic drugs) was plotted as a linear function of drug concentration. The 50% inhibitory concentration (IC_50_) was identified as a concentration of drug required to achieve a 50% growth inhibition relative to untreated controls.

## 3. Results and Discussion

### 3.1. Isolation of CD44v3^high^ALDH^high^ Cells [Cancer Stem Cell- (CSC-) Like Cells] from Human HNSCC Cell Lines

**Figure 1:**
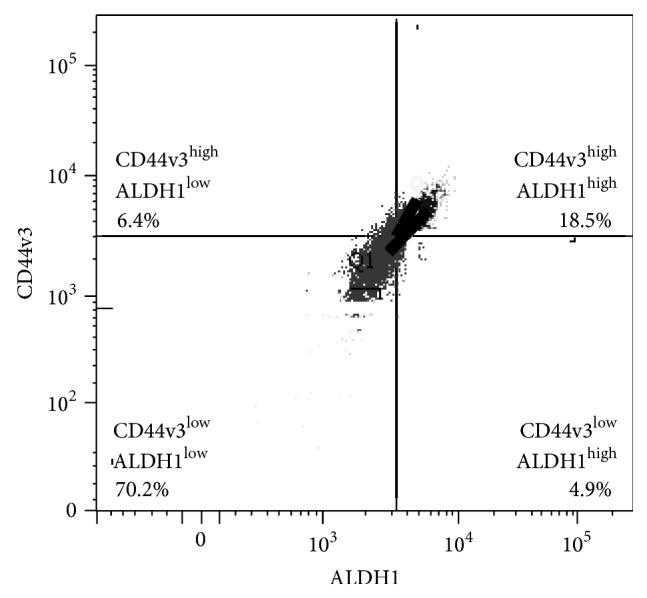
Isolation of cancer stem cell-like population from tumor-derived HNSCC (HSC-3 cells) using multicolor fluorescence-activated cell sorter (FACS). Tumor-derived human HNSCC (HSC-3 cells) were incubated with both ALDEFLUOR kit (to measure an ALDH1 enzymatic activity) and allophycocyanin- (APC-) labeled anti-CD44v3 antibody (recognizing the v3-specific domain of CD44) followed by FACS and cell sorting (Figure 1). Flow cytometry analyses of HSC-3 tumor cell populations including CD44v3^high^ALDH1^high^ (top right quad, 18.5%) or CD44v3^high^ALDH1^low^ (top left quad, 6.4%) or CD44v3^low^ALDH1^high^ (bottom right quad, 4.9%) or CD44v3^low^ALDH1^low^ (bottom left quad, 70.2%). Live tumor cell sorting was then done using a FACS-fluorescence-activated cell sorter to isolate CD44v3^high^ALDH1^high^ cells or CD44v3^low^ALDH1^low^ for the study.

Overexpression of CD44v3 has been shown to be closely associated with HNSCC development and progression. Aldehyde dehydrogenase-1 (ALDH1), a detoxifying enzyme responsible for the oxidation of intracellular aldehydes, is also considered to be a common marker for both normal stem cells and malignant cancer stem cells (CSCs) from HNSCC [15–17]. In this study we isolated CD44v3^high^ALDH1^high^ subpopulations from tumor-derived human HNSCC cells (HSC-3) using FACS-fluorescence-activated cell sorting procedures (Figure 1). Our results indicate that CD44v3^high^ALDH1^high^ cells (to a much lesser extent CD44v3^high^ALDH1^low^, CD44v3^low^ALDH1^high^, CD44v3^low^ALDH1^low^, or unsorted cells) were capable of forming large size tumors in NOD/SCID mice injected with as few as 50 CD44v3^high^ALDH^high^ cells (Table 1). These findings indicate that CD44v3^high^ALDH1^high^ cells display cancer stem cell- (CSC-) like properties by exhibiting very high tumor initiation potential. However, the cellular and molecular mechanisms that produce CSC-like properties of CD44v3^high^ALDH1^high^ cells were not known and, therefore, they are the focus of this investigation.

### 3.2. Analyses of Stemness Marker (Nanog, Oct4, Sox2, and KLF-4) Expression, Cell Growth/Self-Renewal, and Clone Formation (Differentiation) in CD44v3^high^ALDH1^high^ (CSC-Like) Cells following Different Sizes of HA Treatment

Identification of the extracellular matrix (ECM) components [e.g., hyaluronan (HA)] contributing to CSC-like properties will enable us to gain a better understanding regarding the role of the microenvironment in initiating and maintaining CSC properties. The question of whether different sizes of HA fragments (ranging from 5 kDa to 700 kDa) regulate CSC signaling and function in head and neck cancer cells has not been fully addressed and therefore is the focus of this study.

**Figure 2:**
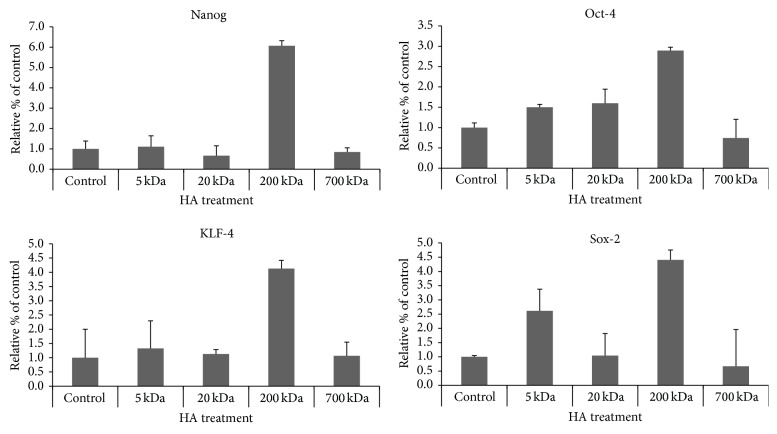
Effects of various sizes of HA on stimulating stem cell marker (Nanog, Oct4, Sox2, and KLF-4) expression in CD44v3^high^ALDH1^high^ cells using Q-PCR analyses.

There is compelling evidence showing that certain stem cell markers such as Nanog, Oct, Sox2, and KLF-4 are known to form a self-organized core of transcription factors that maintain pluripotency and self-renewal of human embryonic stem cells [18–21]. In this study we found that the expressions of Nanog, Oct, Sox2, and KLF-4 are significantly increased in CD44v3^high^ALDH1^high^ cells treated with 200 kDA-HA based on Q-PCR analyses (Figure 2). There is only a low level of stem cell marker expression detected with other sizes of HAs (e.g., 5 kDa-HA, 20 kDa-HA or 700 kDa-HA, or no HA treatment) (Figure 2). These findings clearly indicate that the stem cell markers (Nanog, Oct4, Sox2, and KLF-4) are upregulated in the CD44v3^high^ALDH1^high^ cell subpopulation isolated from HNSCC (HSC-3) following 200 kDa-HA treatment.

**Figure 3:**
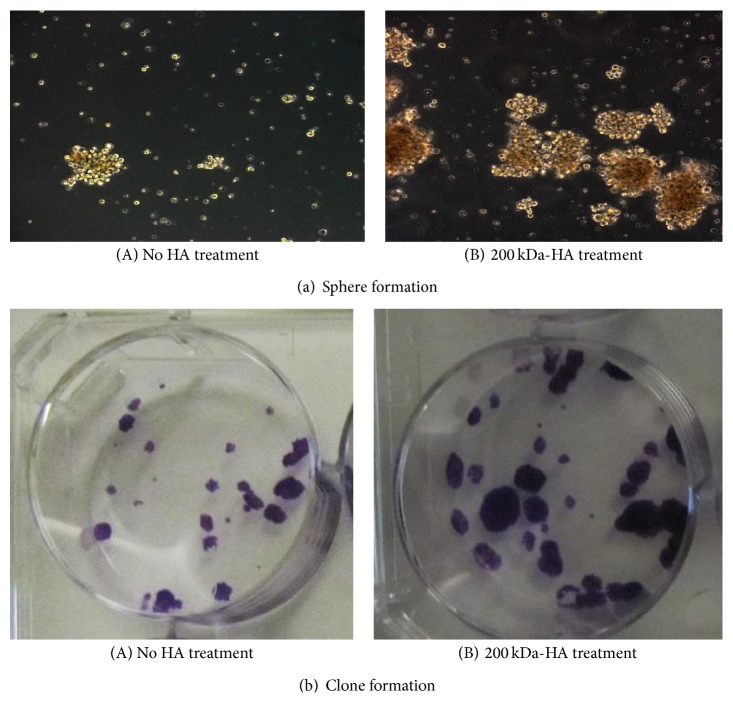
Measurement of sphere formation (a) and clone formation (b) CD44v3^high^ALDH1^high^ (CSC) cells. (a) Sphere formation of CD44v3^high^ALDH1^high^ cells [treated with no HA (A) or with 200 kDa-HA (B)] in a mixture of 5 mg/mL of matrigel (Corning) and defined medium (RPMI-1640 medium containing EGF and bFGF without serum) for 3 weeks (21 days) as described in Section 2. (b) Clone formation (differentiation) of CD44v3^high^ALDH1^high^ cells pretreated with no HA (A) or with 200 kDa-HA (B). Specifically, clone formation and differentiation were induced by incubating CD44v3^high^ALDH^high^ cells (dissociated from spheres treated with 200 kDa-HA or without HA for 10 days as described above). After the removal of 200 kDa-HA from the cells, these sphere-derived CD44v3^high^ALDH^high^ cells were then incubated in RPMI 1640 complete culture and 10% fetal bovine serum for ~7–10 days. After most cell clones expand to >50–100 cells, they were fixed with methanol followed by staining with crystal violet to visualize clone formation as described in Section 2.

To determine whether 200 kDA-HA-treated CD44v3^high^ALDH1^high^ cells (overexpressing stem cell markers, Nanog, Oct4, Sox2, and KLF-4) are capable of undergoing self-renewal and long-term tumor cell growth, we assessed the ability of these tumorigenic CD44v3^high^ALDH1^high^ cells to grow in a “sphere forming” culture by incubating them in serum-free spheroid medium containing 200 kDa-HA (or no HA). After 14 days of incubating these cells in the serum-free medium, we observed that the 200 kDa-HA-treated CD44v3^high^ALDH1^high^ cells form large numbers of spheres (Figure 3(a)-(B)), ranging from 50 to 100 cells per spheroid (Figure 3(a)-(B)). In contrast, only a very small number of spheres were detected in those cells that were not treated with HA (Figure 3(a)-(B)). Therefore, it appears that sphere formation with CD44v3^high^ALDH1^high^ cells involves the binding of 200 kDa-HA.

To further test the ability of these CD44v3^high^ALDH1^high^ cells (untreated or pretreated with 200 kDa-HA followed by dissociation from spheres after a serial passage of 1st, 2nd, and 3rd generation of sphere formation) to undergo clone formation and differentiation, we conducted clone formation assay (Figure 3(b)). Our data showed that the level of clone formation appears to be significantly higher in these 200 kDa-HA-treated CD44v3^high^ALDH1^high^ cells (Figure 3(b)-(B)) as compared to those detected in CD44v3^high^ALDH1^high^ cells with no HA treatment (Figure 3(b)-(A)). These observations suggest that CD44v3^high^ALDH1^high^ cells are capable of displaying cancer stem cell-like properties (e.g., sphere formation, cell growth/self-renewal, and clone formation/differentiation) in a 200 kDa-HA-specific manner.

### 3.3. Analyses of microRNA Production in CD44v3^high^ALDH1^high^ (CSC-Like) Cells following Different Sizes of HA Treatment

**Figure 4:**
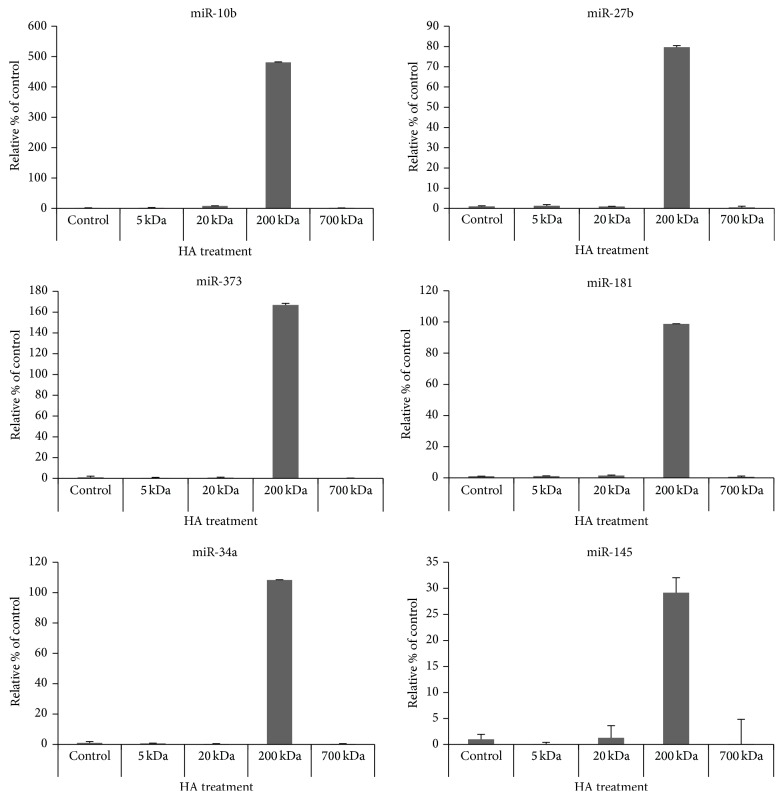
Detection of various miRNA expressions in CD44v3^high^ALDH^high^ cells treated with different sizes of HA. Detection of miR-10b, miR-27b, miR-373, miR-181, miR-34a, and miR-145 production in CD44v3^high^ALDH^high^ cells following the treatment with different sizes of HA (e.g., 5 kDa-HA, 20 kDa-HA, 200 kDa-HA, 700 kDa-Ha, and no HA) and analyzing by Q-PCR as described in Section 2.

**Figure 5:**
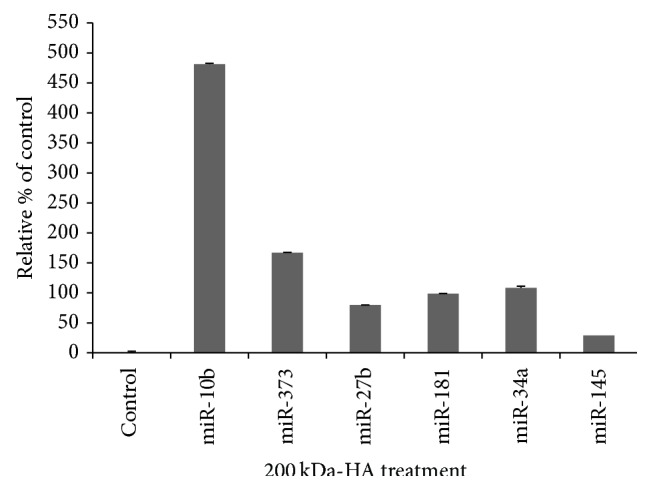
Comparison of miR-10b expression with various other miRNA productions in CD44v3^high^ALDH^high^ cells following the treatment with different sizes of HA (e.g., 5 kDa-HA, 20 kDa-HA, 200 kDa-HA, 700 kDa-HA, and no HA) and analyzing by Q-PCR as described in Section 2.

Accumulating evidence now indicates that noncoding microRNAs (miRNAs, approximately 22 nucleotides) are involved in head and neck cancer development [22–25]. Our previous study demonstrated that certain oncogenic microRNAs promote the cancer stem cell functions of CD44v3^high^ALDH1^high^ (CSC-like) subpopulation from HNSCC (HSC-3) [15]. In this study we found that a panel of stem cell-related miRNAs including miR-10b, miR-27b, miR-373, miR-181, miR-34a, and miRNA-145 is preferentially upregulated by 200 kD-HA. In contrast, the other sizes of HA (e.g., 5 kD-HA, 20 kD-HA, and 700 kDa-HA) fail to induce various miRNA productions in the CD44v3^high^ALDH1^high^ (CSC-like) cell subpopulation (Figure 4). Most noticeably, miR-10 appears to undergo the highest level of stimulation by 200 kDa-HA (Figure 5). In order to verify whether the 200 kDa-HA-induced miRNA-10b contributes to malignancy in the head and neck cancer cells, the following miR-10-regulated functional events were performed.

### 3.4. Detection of Survival Protein Expression and Chemotherapy Resistance

**Figure 6:**
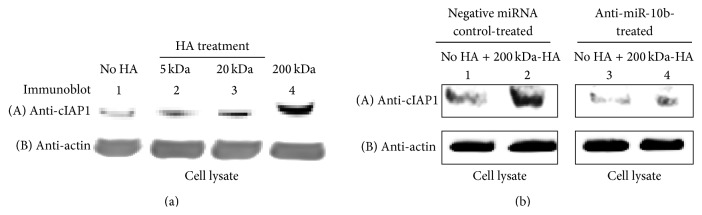
Analyses of 200 kDa-HA-mediated survival protein (cIAP1) expression in CD44v3^high^ALDH^high^ cells. (a) Detection of the expression of cIAP1 by anti-cIAP1-mediated immunoblotting using cell lysate isolated from CD44v3^high^ALDH^high^ cells treated with different sizes of HA [e.g., no HA ((A), lane 1), 5 kDa-HA ((A), lane 2), 20 kDa-HA ((A), lane 3), and 200 kDa-HA ((A), lane 4)] for 3 days. (b) Detection of the expression of cIAP1 by anti-cIAP1-mediated immunoblotting using cell lysate isolated from CD44v3^high^ALDH^high^ cells transfected with negative miRNA control [treated with no HA ((A), lane 1) or with 200 kDa-HA for 24 h ((A), lane 2)] or treated with anti-miR-10b inhibitor with no HA ((A), lane 3) or with 200 kDa HA addition for 3 days ((A), lane 4). The amount of actin detected by anti-actin-mediated immunoblot ((a): (B), lane 1–4; (b): (B), lane 1–4) in each gel lane was used as a loading control.

Survival proteins such as inhibitors of apoptosis proteins (IAPs) are frequently upregulated in CSCs following HA treatment [15]. Importantly, high levels of IAPs in CSCs increase cell survival due to the binding of IAPs to caspases and the suppression of apoptosis [26]. Here, we found that the expression of IAPs such as c-IAP-1 significantly increased in CD44v3^high^ALDH1^high^ cells treated with 200 kDA-HA based on anti-c-IAP-1-mediated immunoblot analyses (Figure 6). There is only a relatively low level of c-IAP-1 expression detected with other sizes of HAs (e.g., 5 kDa-HA, 20 kDa-HA, or no HA treatment) (Figure 6(a)). These findings clearly indicate that the survival proteins such as c-IAP-1 are upregulated in the CD44v3^high^ALDH^high^ cell subpopulation isolated from HNSCC (HSC-3) following 200 kDa-HA treatment.

Our present data also demonstrate that downregulation of miR-10b by treating CD44v3^high^ALDH1^high^ cells with an anti-miR-10b inhibitor (but not a negative-control miRNA) results in the downregulation of cIAP-1 (Figure 6(b), lane 3 and lane 4 versus lane 1 and 2) in the presence of 200 kDa-HA. Together, these results indicate that the signaling network containing miR-10b is functionally coupled to the stimulation of survival protein production in CSCs. These specific effects may facilitate the CSC-mediated HNSCC progression following HA treatment.

**Figure 7:**
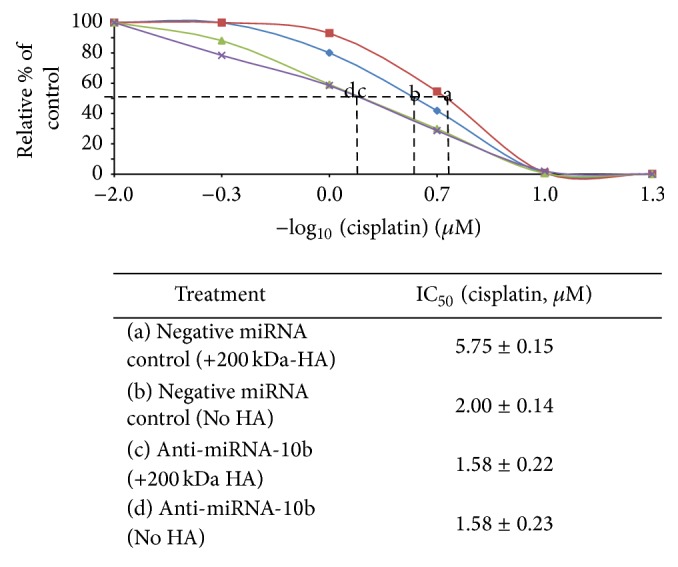
Effects of anti-miR-10b on cisplatin-induced cell growth inhibition in CD44v3^high^ALDH1^high^ cells following 200 kDa-HA treatment. Effects of cisplatin-induced cell growth inhibition in CD44v3^high^ALDH1^high^ cells transfected with negative miRNA control plus 200 kDa-HA addition (a) or with no HA addition (b) or transfected with anti-miR-10b inhibitor plus 200 kDa HA addition (c) or with no HA addition (d) for 7 days. Tumor cell growth inhibition (IC_50_) is designated as “the *μ*M concentration of chemotherapeutic drug (e.g., cisplatin treatment) that causes 50% inhibition of tumor cell growth” using CellTiter-Glo Luminescent Cell Viability Assay as described in the Section 2. IC_50 _values are presented as the means ± standard deviation.

Further analyses indicate that the addition of 200 kDa-HA in negative miRNA-treated CD44v3^high^ALDH1^high^ cells significantly decreases the ability of cisplatin to induce tumor cell death (Figure 7(a) versus Figure 7(b); Table 2). These observations strongly suggest that 200 kDA-HA causes both a decrease in tumor cell death and an increase in tumor cell survival leading to the enhancement of chemoresistance (Figure 7(a) versus Figure 7(b); Table 2). Moreover, downregulation of miR-10b by treating CD44v3^high^ALDH1^high^ HNSCC cells with an anti-miR-10b inhibitor (but not negative miRNA control-treated samples) effectively attenuates 200 kDA-HA-mediated tumor CD44v3^high^ALDH1^high^ cell survival (Figure 7(c) versus Figure 7(d); Table 2) and enhances cisplatin sensitivity in CD44v3^high^ALDH1^high^ cells (Table 2). These findings clearly indicate that downregulation of the 200 kDa-HA-induced miR-10b function (by anti-miR-10b treatment) may represent a new target for therapeutic agents designed to cause head and neck cancer CSCs to undergo cell death and remain chemotherapy sensitive.

## 4. Conclusion

Advanced head and neck squamous cell carcinoma (HNSCC) is an aggressive disease and a deadly cancer. Thus, clarification of key aspects of tumor cell functions underlying the clinical behavior of HNSCC is greatly needed. Cancer stem cells (CSCs) found in head and neck cancer have been implicated in the initiation/development of malignancy [27]. Because little is known regarding the molecular basis underlying CSC signaling and function, it is important to identify molecule(s) which can be used for predicting the oncogenic potential and possible drug targets for head and neck cancer-derived CSCs.

Hyaluronan (HA) is well-known as one of the major components in extracellular matrices (ECM). HA is often bound to CD44, a ubiquitous, abundant, and functionally important cell surface receptor. Different sizes of HA are known to bind to CD44; and our studies show that they influence a variety of cellular functions. However, our understanding of CD44 interaction with different sizes of HA fragments in cancer stem cells (CSCs) from HNSCC is lacking. In this study we found that size-specific HA (in particular, 200 kDa-HA) plays a pivotal role in the selective activation of CD44v3^high^ALDH1^high^ (CSC-like) functions and microRNA signaling (in particular, miR-10b) required for tumor cell survival, chemoresistance, and head and neck cancer progression. Thus, silencing or inhibiting 200 kDa-HA-activated miRNA-10b may provide important new therapeutic targets to block matrix HA-associated CSC behaviors (e.g., tumor cell survival and chemoresistance) in head and neck cancer.

**Figure 8:**
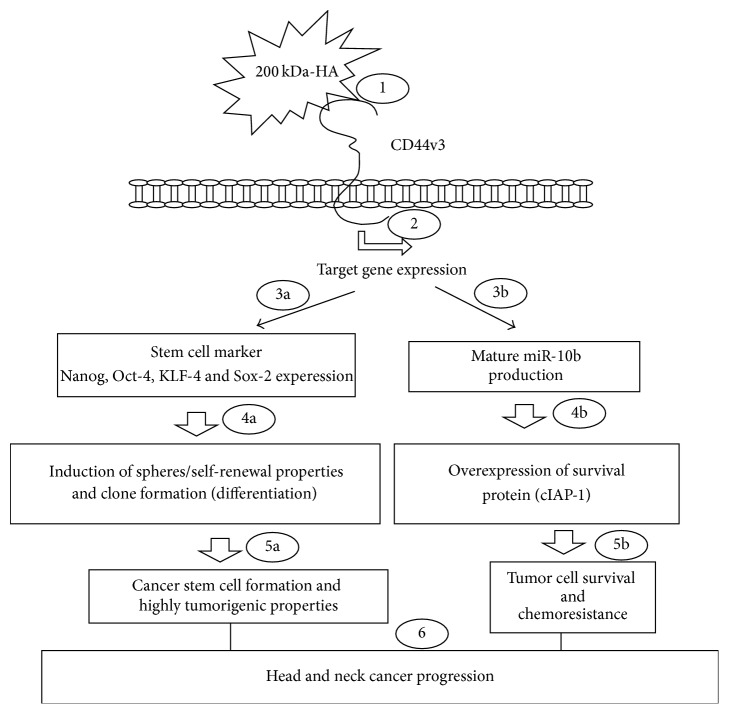
A proposed model for oncogenic signaling induced by 200 kDa-HA in the regulation of miRNA-10 production and cancer stem cell (CSC) functions in CD44v3^high^ALDH^high^ cells. The binding of 200 kDa-HA (step  1) to CD44v3^high^ALDH1^high^ cells promotes specific target gene expression (step  2), including stem cell marker (Nanog, Oct-4, KLF-4 and Sox-2) experession (step  3-a). The resultant stem cell marker expression then induces spheres/self-renewal properties and clone formation (differentiation) (step  4-a) contributing to cancer stem cell formation and highly tumorigenic properties (step  5-a). At the same time, the binding of 200 kDa-HA to CD44v3^high^ALDH1^high^ cells also stimulates miR-10b gene expression/mature miR-10b production (step  3-b) which then stimulates survival protein, IAP (c-IAP1) expression (step  4-b), and HNSCC cell antiapoptosis/survival as well as chemoresistance (step  5-b). Taken together, these findings suggest that HA- (in particular, 200 kDa-HA-) mediated cancer stem cell (CSC) pathways and miR-10b function play a critical role in promoting tumor formation and chemoresistance leading to head and neck cancer progression (step  6).

As summarized in Figure 8, we propose that the binding of 200 kDa-HA (step  1) to CD44v3^high^ALDH1^high^ cells promotes specific target gene expression (step  2), including stem cell marker (Nanog, Oct4, Sox2, and KLF-4) expression (step  3-a). The resultant stem cell marker expression then induces spheres/self-renewal properties and clone formation (differentiation) (step  4-a) contributing to cancer stem cell functions and highly tumorigenic properties (step  5-a). At the same time, the binding of 200 kDa-HA to CD44v3^high^ALDH1^high^ cells also stimulates miR-10b gene expression/mature miR-10b production (step  3-b) which then stimulates survival protein, IAP (c-IAP1) expression (step  4-b), and HNSCC cell anti-apoptosis/survival as well as chemoresistance (step  5-b). Taken together, these findings suggest that HA- (in particular, 200 kDa-HA-) mediated cancer stem cell (CSC) pathways and miR-10b function play a critical role in promoting tumor formation and chemoresistance leading to head and neck cancer progression (step  6).

## Figures and Tables

**Table 1:** Analyses of tumor formation by CD44v3^high^ALDH1^high^ cells, CD44v3^low^ALDH1^low^ cells, CD44v3^low^ALDH1^high^ cells, or unsorted cells subcutaneously injected into NOD/SCID mice.

Cell populations	Tumor formation^*^		
	5,000 cells injected (8 weeks)	500 cells injected (8 weeks)	50 cells injected (8 weeks)
CD44v3^high^ALDH1^high^ cells	20/20	18/20	16/20
CD44v3^low^ALDH1^high^ cells	3/20	1/20	0/20
CD44v3^low^ALDH1^low^ cells	1/20	0/20	0/20
Unsorted cells	2/20	0/20	0/20

^*^For the tumor cell injection, each mouse was subcutaneously inoculated with CD44v3^high^ALDH1^high^ cells or CD44v3^low^ALDH1^high^ cells or CD44v3^low^ALDH1^low^ cells or unsorted cells with 5,000 cells, 500 cells, or 50 cells as described in the Section 2. The values expressed in the Table 1 represent the number of animals developed tumors/total number of animals used in the study. The tumor formation assay was performed on at least 5 different experiments with a standard deviation less than ±5%.

**Table 2:** Effects of anti-miR-10b on cisplatin-induced cell growth inhibition in CD44v3^high^ALDH1^high^ cells following 200 kDa-HA treatment.

Treatments	Cisplatin-induced tumor cell growth inhibition IC_50_ (*µ*M)^*^ (% of control)
Negative miRNA control-treated cells (control) (No HA)	2.00 ± 0.15 (100.00%)
Negative miRNA control-treated cells (+200 kDa-HA)	5.75 ± 0.14 (288.00%)
Anti-miR-10-treated cells (No HA)	1.58 ± 0.22 (0.79%)
Anti-miR-10b-treated cells (+200 kDa-HA)	1.58 ± 0.23 (0.79%)

^*^Tumor cell growth inhibition (IC_50_) is designated as “the *µ*M concentration of chemotherapeutic drug (e.g., cisplatin treatment) that causes 50% inhibition of tumor cell growth” using CellTiter-Glo Luminescent Cell Viability Assay as described in Section 2. IC_50_ values are presented as the means ± standard deviation. All assays consisted of at least six replicates and were performed on at least 3–5 different experiments.
